# Geogenic and anthropogenic sources identification and ecological risk assessment of heavy metals in the urban soil of Yazd, central Iran

**DOI:** 10.1371/journal.pone.0260418

**Published:** 2021-11-29

**Authors:** Somayeh Soltani-Gerdefaramarzi, Mohsen Ghasemi, Behzad Ghanbarian

**Affiliations:** 1 Faculty of Agriculture and Natural Resource, Ardakan University, Ardakan, Iran; 2 Department of Geology, Kansas State University, Manhattan, Kansas, United States of America; Universidade Federal do Rio Grande - FURG, BRAZIL

## Abstract

Urban soil pollution with heavy metals is one of the environmental problems in recent years, especially in industrial cities. The aim of this study is to evaluate the role of geogenic and anthropogenic sources in the urban soil pollution in Yazd, Iran. For this purpose, 30 top-soil (0–10 cm) samples from Yazd within an area of 136.37 Km^2^ and population of nearly 656 thousand are collected, and the concentration of heavy elements is measured. To evaluate factors affecting the concentration of heavy elements in urban soils and determine their possible sources, Multivariate statistical analysis, including correlation coefficient, principal components analysis (PCA) and cluster analysis (CA) are performed. Enrichment Factor (EF), Geo-accumulation index (I_geo_), and Modified potential ecological Risk Index (MRI) are used to assess the level and extension of contamination. Results of this study suggest that As, Cd, Pb and Zn are affected by anthropogenic source, while the concentrations of Fe, Mn, Ni, Cr, Co, Cu and Cs have come from mostly natural geologic sources. As, Cd and Pb are considerably enriched in the area, provided moderately enriched for the elements Mn, Zn and Cu. However, the other heavy elements show minimal enrichment. I_geo_ reveal that Co, Cr, Cs, Cu, Fe, Mn, Zn and Ni with negative values are unpolluted, Pb posed unpolluted to moderately polluted, and As and Cd represent high polluted. Based on the results of the ecological risk factor, the heavy metals of Mn, Ni, Cr, Zn and Cu have a low ecological risk level. More specifically, we find that Pb shows a moderated ecological risk in 39% of the urban soil in the studied area. As and Cd with respectively 100 and 72% contribution have considerable and very high ecological risk. According to the results of MRI, the area is in a very high ecological risk level, and appropriate management practice is essential to reduce the pollution of heavy elements in this area.

## Introduction

The increasing expansion of cities in size and population, rapid growth of urbanization, and development of industry and agriculture have caused many environmental impacts all around the world. Soils are indeed natural purifiers, in addition to supply food. Accordingly, soil pollution is an important environmental hazard that needs to be addressed. Industrial activities pollute and accumulate heavy metals in soils. This pollution significantly reduces the quality of the environment and threatens human health [1,2]. Soil contamination with heavy metals is a global problem and a serious threat to humans, natural ecosystems, water resources and facilities [3,4]. The presence of heavy metals in the human body can cause many problems. The presence of these metals in small amounts in the body may preserve cells (Fe, Zn, Cu, Cr, etc.). However, the presence of more than these metals may cause damage to plant and animal organisms [5].

Heavy metals have two main sources: (1) natural or geogenic, and (2) anthropogenic sources. Natural sources include the erosion of parent rocks and the entry of these metals into soils [6]. However, numerous studies have shown that anthropogenic sources are mostly due to increased industry, traffic and mining in urban areas [7,8]. For example, the highest concentration of Pb in soils is mainly in the vicinity of busy roads [9]. It was shown that the concentration of Pb, Cd and Cu in an old industrial town in northern China exceeded the background level, and the high concentration of these metals along with Zn and Hg metals was considered due to human activities [10]. In another study, It was indicated that Tl, Hg and As in sulfide mineral areas were highly enriched due to the scattered mineral activities in soils under various land uses [11].

One of the common methods for assessing the state of soil pollution to heavy metals is the use of pollution indicators. Enrichment Factor (EF), Geo-accumulation index (I_geo_), and Modified Ecological Risk Index (MRI) are some of the criteria that, considering the concentration of elements, show the degree of soil pollution in an area [12]. It was used the Ecological Risk Index and the Pollution Load Index to study heavy metal pollution in Linfen, China [13]. To assess the human and ecological risk of heavy metals in soils under different land-use types in an urban environment of Bangladesh, was used several indicators including pollution load index and potential ecological risk. The soils from all land-use types showed considerable to very high ecological risks [14]. It was examined the risk assessment of heavy metals in road dust in the industrial city of Anshan in northeastern China and determined the source of their formation. Results showed that Zn and Pb elements originated from road traffic, while Cd, Cr, Fe, Mn, Ni and Sb from industrial activities. The ecological risk index revealed that heavy metals in the region had moderate to high pollution potential [15].

The city of Yazd, located in Yazd province and the center of Iran, is along the main highway connecting north to south. On the other hand, this province is the second producer of greenhouse production in the country, and the tile, ceramic and steel industries in this province have grown a lot in recent years. Due to the industrial situation, greenhouse cultivations and urban traffic and the predictability of high concentrations of heavy metals in the area’s atmosphere, determining the concentration of heavy elements in the soil of this city seems necessary. Although was evaluated the heavy metals contamination of Zn, Pb, Cr, Cu, Ni, As, Co and Cd in urban surface soil in Yazd city [4] and was investigated the most important physical, chemical and mineralogical properties of atmospheric dust deposited and surface soil on Yazd city [16] but human or natural source of heavy metals has not yet been investigated in this area. The objectives of this study are to (1) assess the degree of surface soil pollution in the urban area to heavy metals, (2) estimate the ecological risk of the area by calculating the Modified potential ecological Risk Index and its spatial distribution, and (3) identify the human or natural source of heavy metals in this area.

## Materials and methods

### Study area

**Fig 1:**
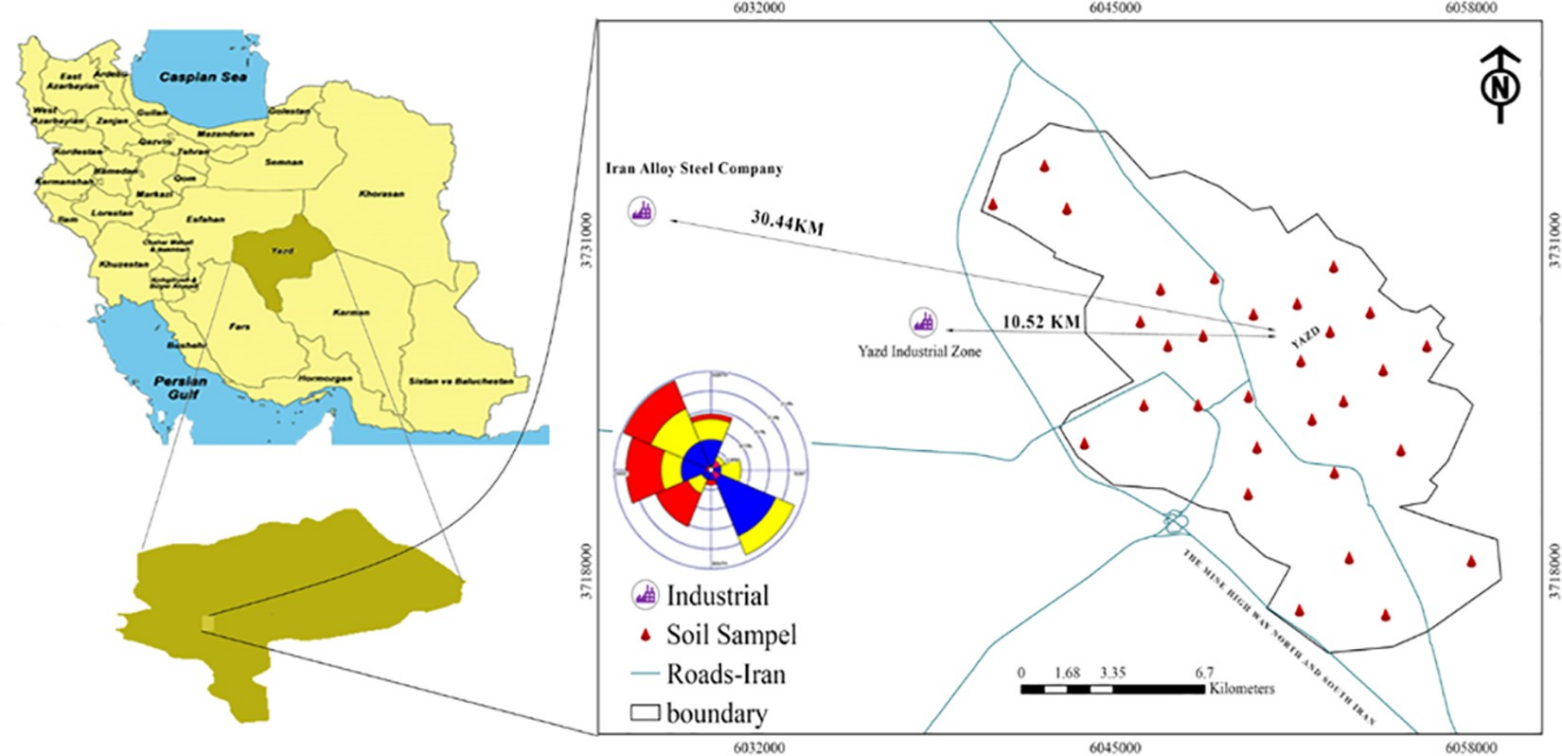
The position of Yazd province in Iran and the urban soil sampling points in Yazd city.

This study was conducted in Yazd, the most populous city in center of Yazd province with an area of 136.37 km^2^ (latitude: 31° 46ʹ to 31° 58ʹ north and longitude: 54° 16ʹ to 54° 26ʹ east) with altitude of 1216 meters above sea level. The prevailing wind directions are northwest from spring to summer, southeast from November to February and west from March and October. The average annual temperature is 19.1°C, the average relative humidity is 31%, and the total annual rainfall is 60.8 mm. According to the last census in 2016, the population of this city is 656,474 people. The primary industry in Yazd province is the ceramic, tile and steel industry. Yazd Industrial Zone and Iran Alloy Steel Company in the west of this town are located at a distance of about 10 km and 30 km from the city center, respectively, and due to the prevailing wind direction [17] in the area, high concentrations of heavy metals in the soil of this region are expected. Due to the use of minerals as a raw material in the preparation of ceramic and tile, the resulting wastewater contains large amounts of salts, minerals and heavy metals. Most of the effluent from the process of producing ceramic and tiles is a gypsum-like liquid that contains large amounts of metals, including the heavy metals Pb, Br, Zr and Fe [18,19]. Also, the concentrations of Pb, Fe, As and Cd are more affected by steel complexes [20,21]. Fig 1 shows the position of Yazd city in Iran and surface soil sampling points in Yazd city.

### Urban soil sampling

Top-soil (0–10 cm) samples were collected in the winter of 2019 from 30 locations in the city. Samples were then dried in room air for 2 days before analysis and next passed through a 200-mesh sieve. The four-acid method (HNO3 + HF + HClO4 + HCl) was used to digest soil samples and then read by ICP-MS Perkin-elmer model [22,23]. Next, total concentrations of As, Cd, Co, Cr, Cs, Cu, Fe, Mn, Ni, Pb and Zn were measured.

### Statistical and geostatistical analysis

The collected data were analyzed using the SPSS package version 16.0 for Windows. Comparison of the mean of the studied parameters and the significance of their differences using Duncan’s test was performed at the 5% level. After determining the initial statistical information, the Kolmogorov-Smirnov test was performed to check the normal distribution of the data. To determine the correlation between the concentration of heavy metals in the soil, Pearson correlation coefficient was used and the box diagrams of the studied parameters were plotted. To evaluate the factors influencing the concentration of heavy soil elements and to determine their possible sources, correlation coefficient analyzes, principal component analysis (PCA) with a varimax rotation and Kaiser Normalization, Cluster Analysis (CA) by Ward’s method and Euclidean distance and Enrichment Factor with consideration of the concentration of the studied elements was performed. Spatial distribution maps of the studied parameters were plotted using inverse distance weighting method (IDW) in ArcGIS 10 (Esri, Redlands, CA, USA).

### Pollution level assessment methods

To assess the level of pollution of heavy metals in urban soils, Enrichment Factor (EF), Geo-accumulation index (I_geo_), and Modified potential ecological Risk Index (MRI) in the area were calculated, as described in what follows.

#### Enrichment factor (EF)

EF is used to determine the degree of contamination of heavy elements in the environment. This factor is applied to separate elements derived from human activities and natural processes as well as to evaluate the degree of human activity. In this normalization technique, the element under study is compared to a reference element. The reference element is an element whose concentration in the environment is slightly variable and is not affected by anthropogenic factors [24]. Elements such as Al, Fe, Mn, Si, and Ti are used as reference elements [24]. In this study, the element Ti in soil samples has been used as a reference element. The enrichment factor is calculated for the elements in the dust using Eq (1):

$$EF=\frac{{{(C}_{n}/C_{ref})}_{sample}}{{{(B}_{n}/B_{ref})}_{background}}$$ (1)

where C_n_ and C_ref_ are the concentrations of the element in the sample and the reference element in the study sample, and B_n_ and B_ref_ are the concentration of the desired element and the reference in the background or earth crust, respectively [25]. If EF is close to 1, it indicates that the elements originated from natural resources and crusts. However, EF between 1 and 10 expresses the origin of elements from natural resources and to some extent human resources. EF > 10 indicates elements from unnatural and human resources [26]. Five classes of contamination are listed in Table 1 based on EF.

**Table 1:** Potential ecological risk based on mEr and MRI and Enrichment Factor (EF) levels.

Ecological risk/ Pollution level	MRI	mEr	EF
Low	MRI < 150	mEr< 40	EF < 2
Moderate	150≤ MRI <300	40≤mEr<80	2≤ EF <5
Considerable	300≤ MRI <600	80≤mEr<160	5≤ EF <20
High	600≤ MRI	160≤mEr<320	20≤ EF <40
Very high	-	320≤ mEr	40≤ EF

#### Geo-accumulation index (I_geo_)

I_geo_, first introduced by [27], has been widely used in the study of heavy elements and is calculated using the following relationship:

$$I_{geo}={log}_{2}\left[\frac{C_{n}}{1.5B_{n}}\right]$$ (2)

To correct and reduce the maternal effects of soil and natural fluctuations and to determine very small anthropogenic effects, following [27] the factor 1.5 is used in Eq (2). According to this index [24,28–30], 7 classes of pollution can be defined: (1) Unpolluted (I_geo_ ≤ 0), (2) unpolluted to moderately polluted (0 < I_geo_ < 1), (3) moderately polluted (1 < I_geo_ < 2), (4) moderately to highly polluted (2 < I_geo_ < 3), (5) highly polluted (3 < I_geo_ < 4), (6) highly to very highly polluted (4 < I_geo_ < 5), and (7) very highly polluted (I_geo_ ≥ 5).

#### Modified potential ecological risk index (MRI)

MRI of heavy metals has recently been used in soil and dust pollution studies, especially in arid regions [31–33]. Ecological risks from heavy metals contamination were quantified and assessed using the potential ecological risk factor (mEr) and the modified potential ecological risk index (MRI) is calculated using the following equations:

$${mEr}_{i}=T_{r}\times {EF}_{i}$$ (3)

$$MRI=\sum_{i=1}^{n}{mEr}_{i}$$ (4)

in which mEr is the potential ecological risk factor of each element, MRI is the modified potential ecological risk, Tr is the toxicity factor for a substance, the amount for Cd, As, Cu, Pb, Ni, Cr, Mn and Zn is 30, 10, 5, 5, 5, 2, 1 and 1 respectively [34]. Modified potential ecological risk is classified according to mEr_i_ and MRI value as a Table 1 [35].

## Results and discussion

### Heavy metal concentrations

**Fig 2:**
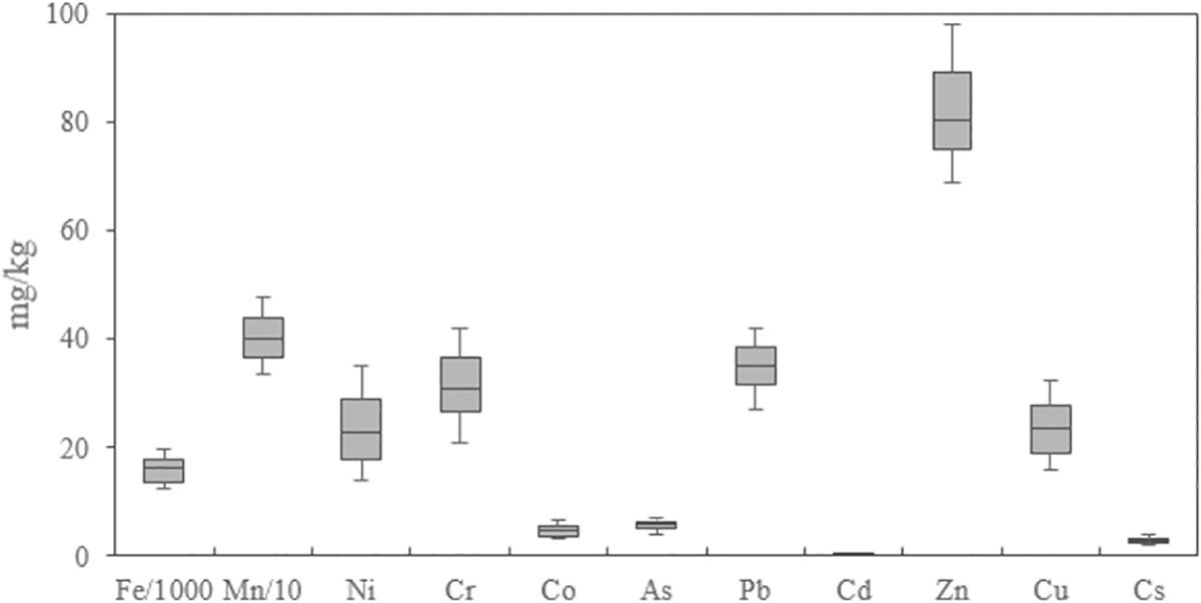
Box-Plot of the heavy metal concentrations in urban soil. (To display all the elements in an axis, the values of Fe and Mn are divided into 1000 and 10, respectively).

The mean concentrations of heavy elements As, Pb, Zn, Cu, Cd, Ni, Cr, Co, Mn, Fe and Cs in surface urban soil samples were 5.86, 34.5, 83.94, 23.50, 0.27, 23.44, 32.61, 4.86, 407.67, 16207 and 2.97 mg/kg, respectively. According to Table 2, the concentrations of As, Cd, Pb and Zn in the studied area are higher than the crust values [25] indicating the possible anthropogenic origin of these elements in urban soil. However, the concentration of other elements include Co, Cr, Cs, Cu, Fe, Mn and Ni are lower than that of the earth’s crust, which could confirm that these elements may be of natural origin [29]. The highest value of As, Pb, Zn, Cu, Cd, Ni, Cr, Co, Mn, Fe and Cs were respectively 8.4, 41, 117, 33, 0.4, 36, 55, 7.6, 548, 22232 and 4.2 mg/kg. The coefficient of variation (CV) of all heavy metals measured was less than 50%, which indicates the low variability of these metals in the urban soil. It has been reported that the CV values of heavy metals from natural sources are relatively low, While CV of heavy metals has high values due to anthropogenic sources [36]. Box-Plot of the heavy metal concentrations in urban soil has been illustrate in Fig 2.

**Table 2:** Descriptive statistics of heavy metal concentrations in urban soils of Yazd city, crust values [23] and Iran standard value [37] (mg·kg^-1^).

	Range	Minimum	Maximum	Mean	SD	CV%	Crust Value	Standard Value
**As**	4.50	3.90	8.40	5.86	1.10	18.77	1.5	18
**Cd**	0.20	0.20	0.40	0.27	0.055	20.37	0.098	2
**Co**	4.50	3.10	7.60	4.86	1.14	23.46	17	40
**Cr**	34.00	21.00	55.00	32.61	8.34	25.57	83	110
**Cs**	2.10	2.10	4.20	2.97	0.53	17.85	4.6	-
**Cu**	17.00	16.00	33.00	23.50	4.80	20.43	25	100
**Fe**	9937.00	12295.00	22232.00	16207	2696.56	16.64	35000	-
**Mn**	214.00	334.00	548.00	407.67	53.23	13.06	600	-
**Ni**	22.00	14.00	36.00	23.44	6.37	27.18	44	50
**Pb**	14.00	27.00	41.00	34.50	4.47	12.96	17	50
**Zn**	48.00	69.00	117.00	83.94	13.60	16.20	71	200

### Multivariate statistical analysis

To evaluate the factors influencing the concentration of heavy metals and determine their possible source, correlation coefficient analyzes, principal component analysis (PCA) [7–24] and cluster analysis (CA) [38,39] were performed by considering the concentration of elements in urban surface soil. The results of correlation analysis presented in Table 3 show that significant positive or negative correlations were found at 1% or 5% level between elements.

**Table 3:** Pearson correlation coefficient matrix for heavy metal concentrations in the urban soil samples.

	As	Cd	Co	Cr	Cs	Cu	Fe	Mn	Ni	Pb
Cd	-0.072									
Co	0.854**	-0.260								
Cr	0.869**	-0.290	0.961**							
Cs	0.803**	-0.237	0.864**	0.924**						
Cu	0.641**	0.022	0.264	0.319	0.307					
Fe	0.841**	-0.197	0.960**	0.924**	0.818**	0.219				
Mn	0.899**	-0.005	0.920**	0.894**	0.791**	0.368	0.911**			
Ni	0.889**	-0.374	0.943**	0.947**	0.906**	0.363	0.919**	0.876**		
Pb	-0.343	0.527*	-0.630**	-0.514*	-0.357	0.187	-0.659**	-0.471*	-0.604**	
Zn	0.098	0.558*	-0.268	-0.124	-0.164	0.454	-0.195	-0.014	-0.230	0.597**

** And * correlation is significant at the 0.01 and 0.05 level.

Cd had the least significant correlation with other heavy metals indicating its different origin in urban soil compared to other elements, and the most significant correlation were observed As and cobalt metals with a large number of elements. Numerous studies have shown that there is a significant correlation between heavy metals with similar source [13,40–42]. It was suggested that the enrichment of heavy metals, such as Cu, Pb and Zn in urban areas confirms the anthropogenic source of these elements [43]. Results of correlation analysis in this study also identify the anthropogenic source for some metals in the studied area. In many studies, traffic is considered to be the source of heavy metals, such as Pb and Zn [44–47]. In this study, we also found that only Zn had a strong correlation with Pb in 1% statistically.

**Fig 3:**
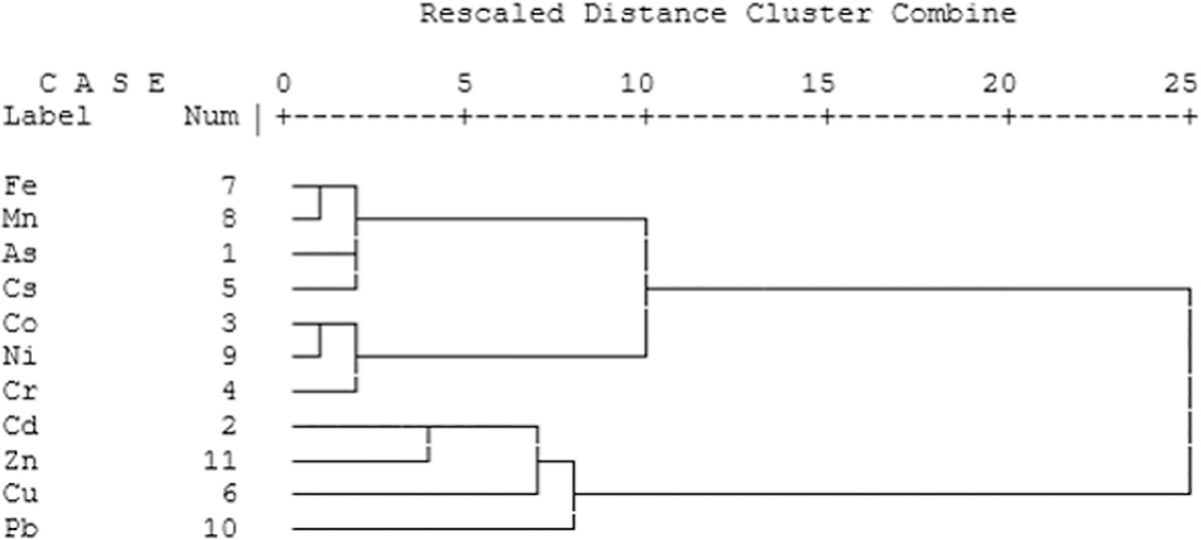
Dendrograms of cluster analysis for heavy metals from Yazd city.

Results of cluster analysis, used to group variables of the same source [13–48], are presented in the dandrogram of Fig 3. In the cluster analysis diagram, the distance between the clusters indicates the degree of relationship between the variables. For example, the low distances show a strong relationship and the high distances indicate a weak relationship between variables. According to the results of cluster analysis, Fe, Mn, As and Cs were placed in a strong cluster and it is suggested a similar source for these elements. The second cluster includes Co, Ni, and Cr, which can be considered the source of industrial activity for this cluster [13–39] and the third group includes the elements of Cd, Zn, Cu and Pb in one cluster, and due to the greater distance than the other two groups in the dendrogram, the relationship of this cluster is relatively weak. It was introduced that the source of vehicle traffic, steel production and fossil fuels for Cu and Pb heavy metals [49].

Results of the principle component analysis after the rotation of the varimax on the data of the concentration of heavy elements in the studied urban soil are presented in Table 4. Two major components were identified with eigenvalues greater than 1 explaining 83.26% of the total variance. Accordingly, 61.11% of system variance was found in PC1, indicated strong (>0.8) positive loading for As, Cr, Co, Cs, Fe, Mn and Ni. PC1 can be better described as a natural origin (natural geochemical processes) and proposing that these metals are from geochemical weathering of the parent rock material.

**Table 4:** Rotated component matrix for the PCA loadings of heavy metals in urban soil of Yazd city.

	Component	
Elements	1	2
As	**0.960**	0.173
Cd	-0.168	**0.699**
Co	**0.950**	-0.249
Cr	**0.961**	-0.148
Cs	**0.895**	-0.109
Cu	0.488	**0.595**
Fe	**0.932**	-0.225
Mn	**0.946**	0.021
Ni	**0.958**	-0.228
Pb	-0.473	**0.744**
Zn	-0.029	**0.901**
Initial eigenvalue	6.913	2.246
% of total variance	61.109	22.152
% of cumulative variance	61.109	83.26

Values in bold refer to factor loadings > 0.5.

PC2 with a variance of 22.15%, showed strong (>0.7) positive loading for Cd, Cu, Pb and Zn. Since the load factor indicates the relationship between the variables and each factor, it can be concluded that the elements As, Cr, Co, Cs, Fe, Mn and Ni have a possible similar source and the heavy metals Cd, Cu, Pb and Zn originate from a alike and different source (anthropogenic sources) from the first group and enter the urban soil. It has been reported that high levels of Pb in urban soils were related with vehicle exhaust emissions from the use of lead gasoline [46]. In addition, the compounds of Zn have been widely used as antioxidants and as a detergent and dispersant of lubricating oils [50]. Therefore, the wear and tear of tires has significantly contributed to the zinc content in urban soils. Copper is used in vehicle braking systems and in car radiators, as a results the destruction of mechanical parts of vehicles over time led to the accumulation of Cu and Zn in urban soils [51]. Therefore, in addition to industrial activities, vehicle emissions can also play a significant role in the accumulation of heavy metals in Yazd’s urban soils.

### Evaluation of heavy metal pollution

**Fig 4:**
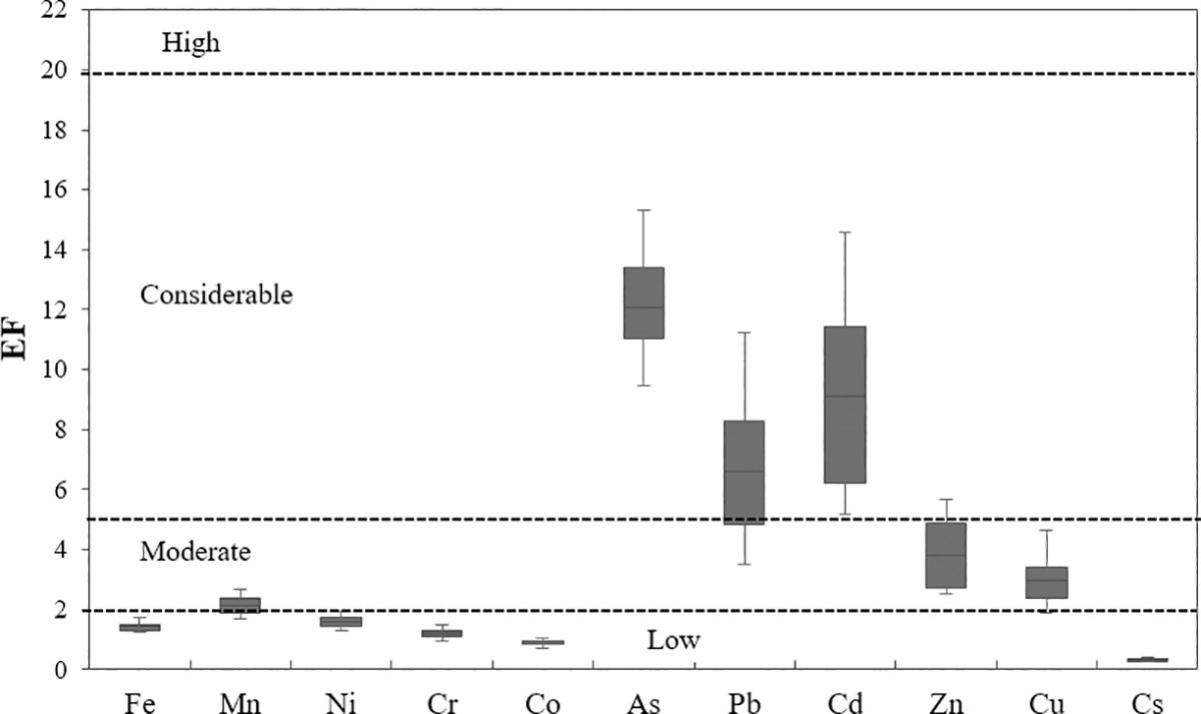
Box-plots of EF for heavy metals in the urban soil samples of Yazd.

Fig 4 shows the values of the EF for heavy metals in urban soil in the studied area. Among the heavy metals investigated, Fe (1.13 to 1.75 with a mean of 1.43), Ni (1.31 to 1.92 with a mean of 1.63), Cr (0.96 to 1.48 with a mean of 19), Co (0.70 to 0.6 with a mean of 0.87) and Cs (0.25 to 0.39 with a mean of 0.30) were set at class 1 (EF < 2) and present low pollution. Mn (1.71 to 2.65 with a mean of 2.09), Zn (2.52 to 5.65 with a mean of 3.33) and Cu (1.87 to 4.65 with a mean of 2.90) were placed in class 2 (2<EF<5) and have been enriched moderately. As (9.48 to 15.32 with a mean of 11.85), Pb (3.31 to 11.22 with a mean of 6.47) and Cd (5.20 to 14.95 with a mean of 9.12) are also included in the level 3(5≤EF<20) and polluted considerably. Generally, the ranking of EF levels of the metals in urban soil was as follows: As > Cd > Pb > Zn > Cu > Mn > Fe > Ni > Cr > Co > Cs.

**Fig 5:**
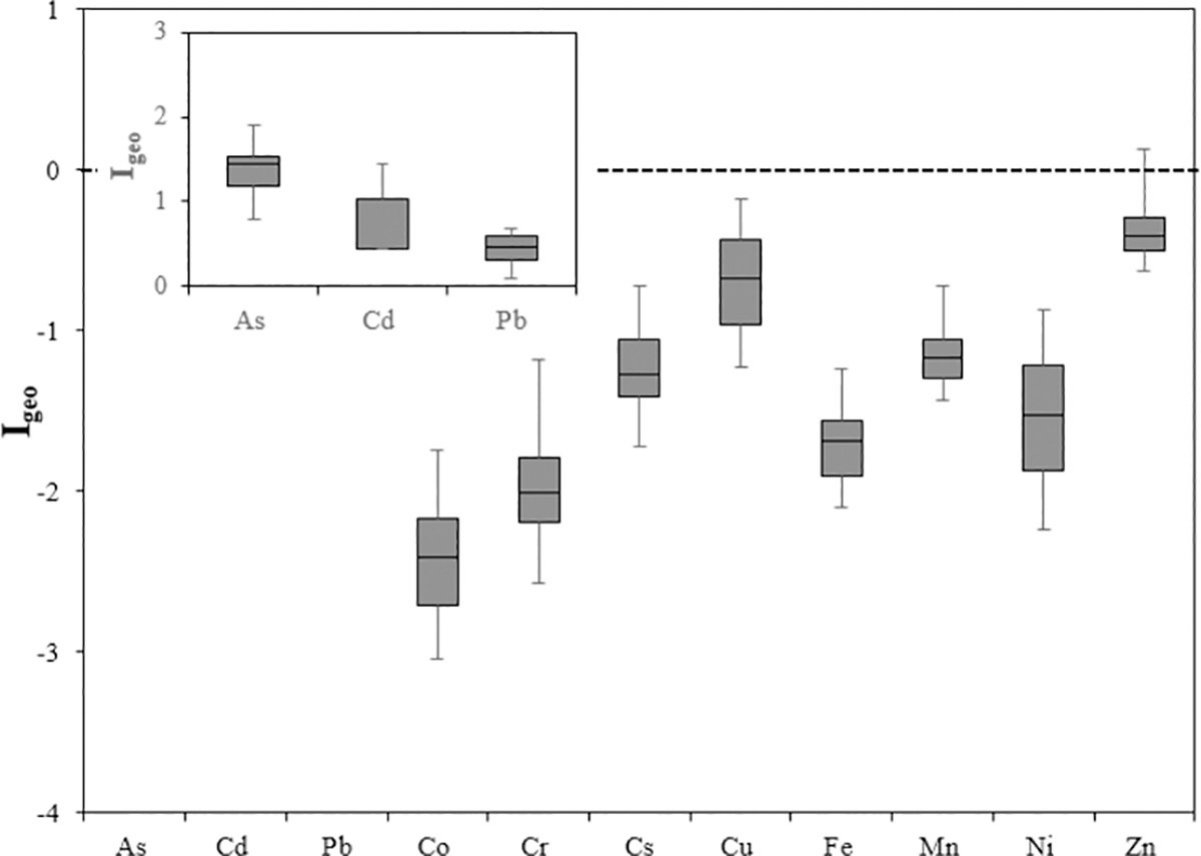
Box-plots of I_geo_ for heavy metals in the urban soil samples of Yazd.

Fig 5 shows the values of the I_geo_ of heavy elements in urban soil. The I_geo_ of 11 elements in urban soil samples varied from -3 to 2, respectively. The lowest and highest were obtained for Co and As. Since the average Geo-accumulation index (I_geo_) was greater than one, a contamination was detected by metals in the study area. The elements Co, Cr, Cs, Cu, Fe, Mn, and Ni with a maximum I_geo_ ≤ 0 have unpolluted. As (0.79 to 1.99 with a mean of 1.44) and Cd (0.44 to 1.44 and a mean of 1.03) were classified as moderately polluted. The Pb element (0.08 to 0.68 and with a mean 0.46) was found in the second class and was unpolluted to moderately pollute. Zn element with a minimum and maximum value of the I_geo_ 0.63 and 0.13 respectively, and with a mean of 0.4 is placed in the unpolluted class, although its maximum value was observed to be more than 1 and it can be considered as a border element. As can be seen from Table 2, the value specified in the earth’s crust for Zn metal is considered to be 71, and its average value in this study was 83.94.

### Evaluation of ecological risk index

**Fig 6:**
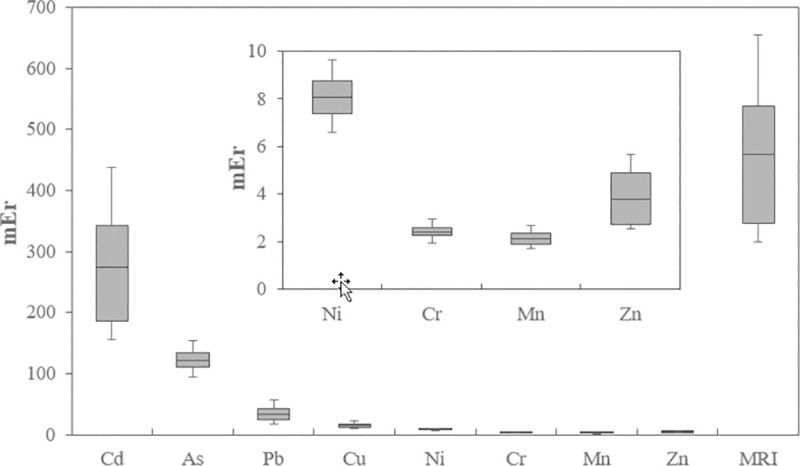
Box-plots of mEr and MRI for heavy metals in the urban soil samples of Yazd.

The box diagram of the ecological risk factor (mEr) and the modified ecological risk index (MRI) of heavy metals in the urban soil samples of the studied area is shown in Fig 6. Based on the obtained results, the elements of Mn (from 1.71 to 2.65 with a mean of 2.12), Ni (6.65 to 6.61 with a mean of 0.87), Cr (1.93 to 2.96 with a mean of 2.6), Zn (2.52 to 5.65 with a mean of 3.79) and Cu (93.33 to 24.24 with a mean of 14.81) determined values less than 40 and are located in the first class or in other words these heavy metal are at low ecological risk level. Pb with a range of 17.67 to 56.08 with a mean of 33.03, on the second level, showed moderated ecological risk. As (94.81 to 153.25 and with a mean 120.77) is located in the range of 80 to 160 and therefore has considerable ecological risk, and Cd with mEr ≥ 320 (155.91 to 437.83 and the mean of 274.10) represent very high ecological risk. In general, the ranking of mEr levels of the metals in urban soil was as follows: Cd > As > Pb > Cu > Ni > Zn > Cr > Mn.

**Fig 7:**
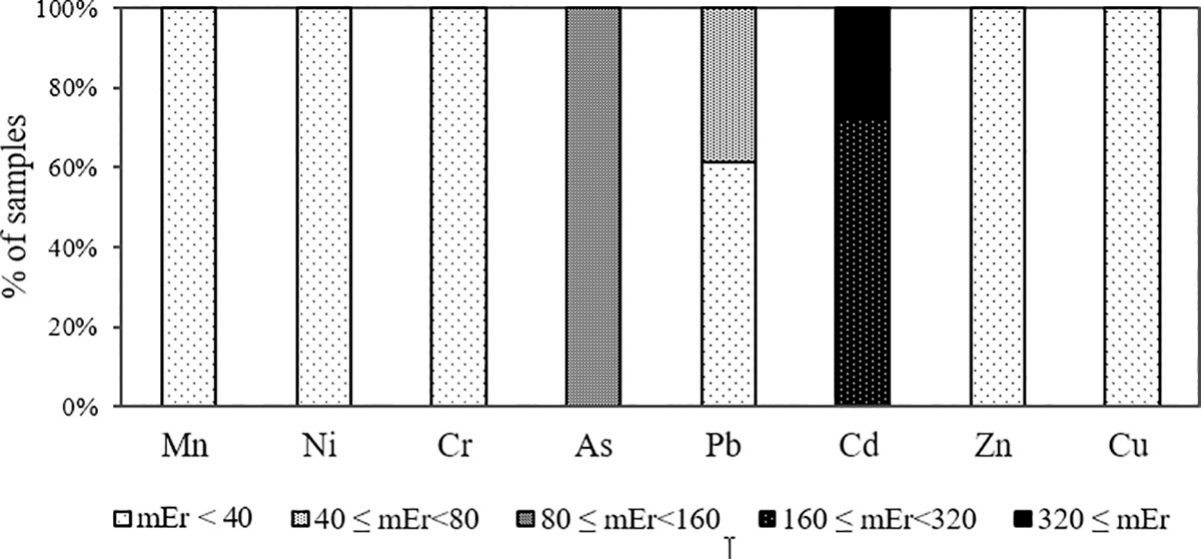
The potential ecological risk factor (mEr) characteristics of heavy metals in the urban soils of Yazd.

The MRI value of heavy metals in the studied urban soil of Yazd ranged from 314.58 to 654.98, which indicates very high ecological risk. According to Fig 7, 100% of the studied area is low in ecological risk for heavy metals of Mn, Ni, Cr, Zn and Cu and have considerable level for As. In the case of Pb, 61% and 39% of the area had low and moderated ecological risk, respectively. Cd ecological risk assessment also showed that 72% and 28% of the studied area were in the high and very high risk class.

**Fig 8:**
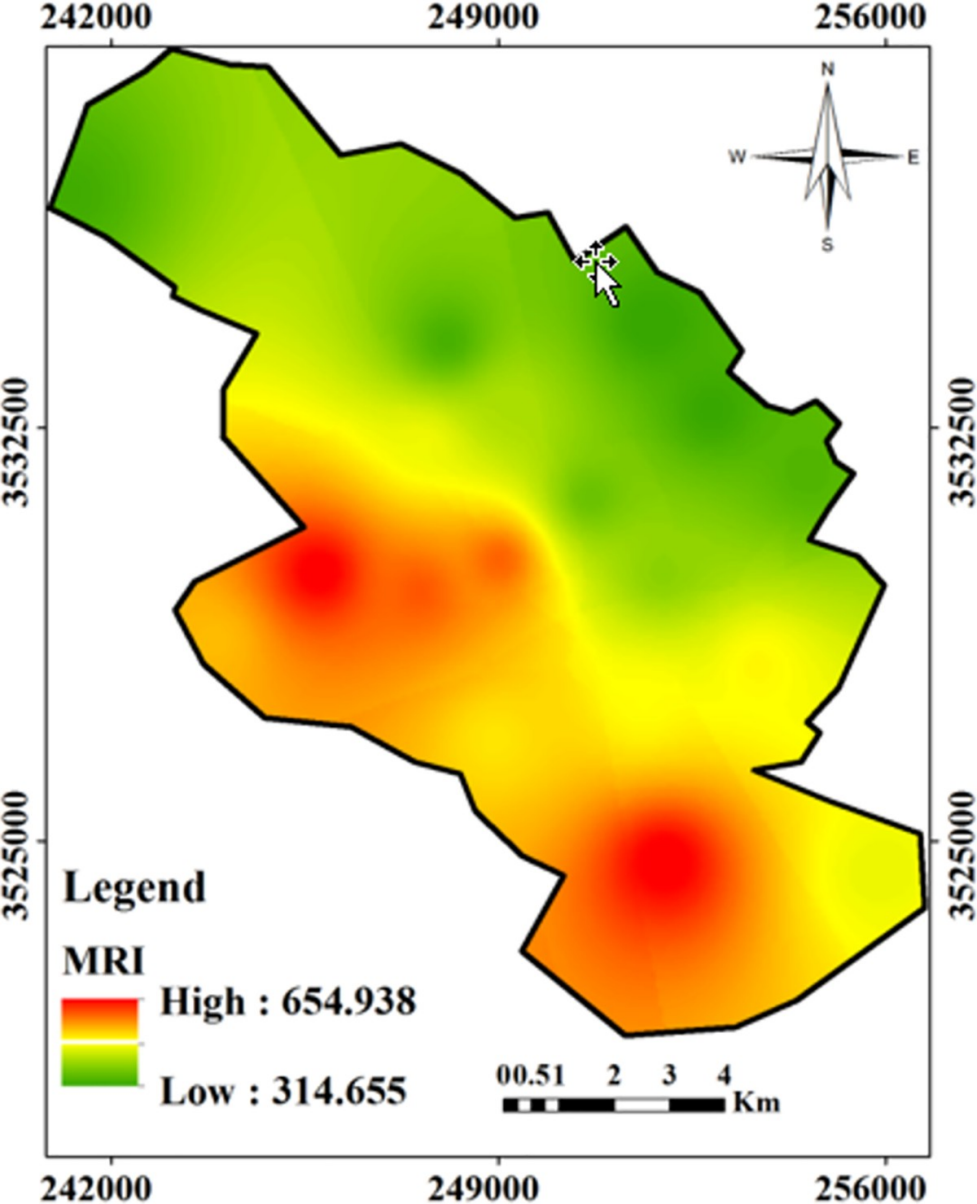
The spatial distribution of the Modified potential ecological risk index (MRI) in urban soil of Yazd city.

The spatial distribution of the Modified potential ecological risk index (MRI) in urban soil of Yazd city has been shown in Fig 8. According to the results, the highest values of MRI were found in the southern and western parts of the city. These areas have a large concentration of population and high traffic.

## Conclusion

Heavy metals in urban area soils can have both geogenic and anthropogenic sources due to industry, industrial effluents, chemical fertilizers [42,52,53]. In this study the concentration of all elements was lower than the standard level of Iranian soil resources (Table 2). Although according to the results, 100% of the studied area is low in ecological risk for heavy metals of Mn, Ni, Cr, Zn and Cu and have considerable level for As. In the case of Pb, 61% and 39% of the area had low and moderated ecological risk, respectively. Cd ecological risk assessment also indicated that 72% and 28% of the studied area were in the high and very high risk class. The results of Pearson correlation analysis, PCA and CA, as well as the results of the I_geo_, showed that the source of heavy metals in the urban soil of the study area can be divided into 3 main categories. The elements Cd, Cu, Pb, and Zn originated from anthropogenic sources including off-road vehicular traffic and industrial activity such as tile, ceramic and steel industries, as the value of these elements was greater than it of the earth’s crust, and the I_geo_ ≥ 1 (Table 1 and Fig 5). On the other hand, Cd had only a significant correlation with Pb and Zn. However, As was an element that has a significant correlation with other elements and was in the same group with other elements according to PCA and CA. It was about 4 times greater than that in the earth’s crust (Table 2) and does not appear to be in the same group as the other elements. Based on the results of this study, it seems that a separate source of anthropogenic is proposed for this element such as fertilization and the use of fossil fuels in steel production due to the proximity of the Alloy Steel Company to the Yazd city center. On the other hand, according to the results of [54], the major sources of arsenic contamination in are gold and copper mines and Urmia-Dokhtar volcanic formation, which contain various heavy and toxic metals. Most of Iran’s mines, including Yazd, are located on this formation, and the use of old mining methods and equipment has increased the intensity of pollution. In addition, the use of pesticides, insecticides and other agricultural inputs has led to arsenic entering these resources. Other elements, including Fe, Mn, Cr, Co, Cs and Ni, are also in the third group, which according to the I_geo_ < 0 and have a natural and geogenic origin. The results of the PCA and CA also confirm these result.

## Supporting information

S1 Data (XLSX)

## References

[1] Ke-Lin HU, Zhang FR, Hong L, Huang F, Bao-Guo LI. Spatial patterns of soil heavy metals in urban-rural transition zone of Beijing. Pedosphere. 2006;16: 690–8.

[2] Islam MS, Ahmed MK, Al-Mamun MH, Islam SMA. Sources and ecological risks of heavy metals in soils under different land uses in Bangladesh. Pedosphere. 2019; 29: 665–75.

[3] Tianlik TEH, Norulaini NARN, Shahadat M, Yoonsing WONG, Omar AKM. Risk assessment of metal contamination in soil and groundwater in Asia: A review of recent trends as well as existing environmental laws and regulations. Pedosphere. 2016; 26: 431–50.

[4] Soltani-Gerdefaramarzi S, Ghasemi M, Gheysouri M. Pollution, human health risk assessment and spatial distribution of toxic metals in urban soil of Yazd City, Iran, Environ Geochem Health. 2021a;1–16. 10.1007/s10653-021-00844-y. 33559784

[5] Zhu YC, Wang LJ, Zhao XY, Lian J, Zhang ZH. Accumulation and potential sources of heavy metals in soils of the Hetao area, Inner Mongolia, China. Pedosphere. 2020; 30:244–52.

[6] Alloway BJ. Heavy Metals in Soils. John Wiley & Sons Inc., New York, 1995; 368 p.

[7] Qu MK, Li WD, Zhang C R, Wang SQ, Yang Y, He LY. Source apportionment of heavy metals in soils using multivariate statistics and geostatistics. Pedosphere. 2013; 23:437–44.

[8] Shangguan YX, Cheng B, Zhao L, Hou H, Ma J, Sun ZJ, et al. Distribution assessment and source identification using multivariate statistical analyses and artificial neutral networks for trace elements in agricultural soils in Xinzhou of Shanxi Province, China. Pedosphere. 2018; 28: 542–54.

[9] Shayler H, McBride M, Harrison E. Sources and Impacts of Contaminants in Soils. Cornell Waste Management Institute. Department of Crop and Soil Sciences. 2009. Cornell University.

[10] Li X, Liu L, Wang Y, Luo G, Chen X, Yang X, et al. Heavy metal contamination of urban soil in an old industrial city (Shenyang) in Northeast China. Geoderma. 2013; 192:50–8.

[11] Ma L, Xiao T, Ning Z, Liu Y, Chen H, Peng J. Pollution and health risk assessment of toxic metal (loid) s in soils under different land use in sulphide mineralized areas. Sci Total Environ. 2020; 138176. doi: 10.1016/j.scitotenv.2020.138176 32247118

[12] Reddy M, Basha S, Sravan Kumar VG, Joshi HV, Ramachandraiah G. Distribution, enrichment and accumulation of heavy metals in coastal sediments of the Alang- Sosiya ship scrapping yard, India. Mar Pollut Bull. 2004; 48:1055–59. doi: 10.1016/j.marpolbul.2003.12.011 15172811

[13] Yang P, Drohan P J, Yang M, Li H. Spatial variability of heavy metal ecological risk in urban soils from Linfen, China. Catena. 2020; 190:104554.

[14] Islam MS, Ahmed MK, Habibullah-Al-Mamun M, Eaton DW. Human and ecological risks of metals in soils under different land use in an urban environment of Bangladesh. Pedosphere. 2020; 30: 201–13.

[15] Xiao Q, Zong Y, Malik Z, Lu S. Source identification and risk assessment of heavy metals in road dust of steel industrial city (Anshan), Liaoning, Northeast China. Hum Ecol Risk Assess. 2019;1–20. doi: 10.1080/10807039.2019.1615828 31404325 PMC6688638

[16] Soltani-Gerdefaramarzi S, Morovati M. The most important physical, chemical and mineralogical properties of atmospheric dust deposited on Yazd city (Central Iran). Phys Geog Res. 2021; 53:21–36. doi: 10.22059/jphgr.2021.311052.1007558 (In Persian with English abstract).

[17] Soltani-Gerdefaramarzi S, Ghasemi M, Ghaneie-Bafghi M. Spatial and temporal Variability in the dust deposition rate of Yazd city and its relationship with some climatic parameters. J Nat Environ. 2021b; 73:701–714. doi: 10.22059/jne.2021.303249.1993 (In Persian with English abstract).

[18] Babu NV, Rao PJ, Prasad IVRKV. Impact of Municipal Solid Waste on Groundwater in the Environs of Greater Visakhapatnam Municipal Corporation Area, Andhrapradesh, India. Int J Eng Sci Invention. 2013; 2: 28–32.

[19] Aljaradin M, Kenneth M P. Environmental impact of municipal solid waste landfills in semi-arid climates-case study–Jordan. J Waste Manag. 2012; 5: 28–39.

[20] Rastmanesh F, Zarosvandi A, Hormozinejad F. An investigation on Khuzestan steel industry in soil pollution around it. First International Congress of Earth Sciences. 2013; Tehran, Iran. (In Persian with English abstract).

[21] Sistani N, Moeinaddini M, Khorasani N, Hamidian A, AliTaleshi M. Azimi Yancheshmeh R. Heavy metal pollution in soils nearby Kerman steel industry: metal richness and degree of contamination assessment. Iran J Health Environ. 2017; 10: 75–86. (In Persian with English abstract).

[22] Amr MA, Helal AFI, Al-Kinani AT, Balakrishnan P. Ultra-trace determination of 90Sr, 137Cs, 238Pu, 239Pu, and 240Pu by triple quadruple collision/reaction cell-ICP-MS/MS: Establishing a baseline for global fallout in Qatar soil and sediments. J Environ Radioact. 2016; 153: 73–87. doi: 10.1016/j.jenvrad.2015.12.008 26736181

[23] Pietilä H, Perämäki P, Piispanen J, Star M, Nieminen T, Kantola M. et al. Determination of low methylmercury concentrations in peat soil samples by isotope dilution GC-ICP-MS using distillation and solvent extraction methods. Chemosphere. 2015; 124: 47–53. doi: 10.1016/j.chemosphere.2014.11.001 25434268

[24] Lu X, Wang L, Lei K, Huang J, Zhai Y. Contamination assessment of copper, lead, zinc, manganese and nickel in street dust of Baoji, NW China. J Hazard Mater. 2009; 161:1058–62. doi: 10.1016/j.jhazmat.2008.04.052 18502044

[25] McLennan SM. Relationships between the trace element composition of sedimentary rocks and upper continental crust. Geochem Geophys. 2001; 2: 1–24.

[26] Wang R, Zou X, Cheng H, Wu X, Zhang C, Kang L. Spatial distribution and source apportionment of atmospheric dust fall at Beijing during spring of 2008–2009. Environ Sci Pollut Res. 2015; 22: 3547–57. 10.1007/s11356-014-3583-3 25249051

[27] Muller G. Index of geoaccumulation in sediments of the Rhine River. GeoJournal. 1969; 2: 108–18.

[28] Guéguen F, Stille P, Geagea ML, Boutin R. Atmospheric pollution in an urban environment by tree bark biomonitoring–Part I: Trace element analysis. Chemosphere. 2012; 86: 1013–19. doi: 10.1016/j.chemosphere.2011.11.040 22169208

[29] Qiang L, Yang W, Jingshuang L, Quanying W, Mingying Z. Grain-size distribution and heavy metal contamination of road dusts in urban parks and squares in Changchun, China. Environ Geochem Health. 2015; 37: 71–82. doi: 10.1007/s10653-014-9631-6 25049053

[30] Tang R, Ma K, Zhang Y, Mao Q. The spatial characteristics and pollution levels of metals in urban street dust of Beijing, China. J Appl Geochem. 2013; 35: 88–98.

[31] Shi Z, Zhang W, Liu Y, Wu F, Dong M, H. et al. PTEs pollution and the ecological risk assessment of urban street dust in Kunming, China. In: IEEE International Geoscience and Remote Sensing Symposium. 2011, Vancouver, BC, Canada.

[32] Kamani H, Mahvi AH, Seyedsalehi M, Jaafari J, Hoseini M, Safari GH. et al. Contamination and ecological risk assessment of PTEs in street dust of Tehran, Iran. Int J Environ Sci Technol. 2017; 14: 2675–82.

[33] Mirzaei-Aminiyan M, Baalousha M, Mousavi R, Mirzaei Aminiyan F, Hosseini H, Heydariyan A. 2018, The ecological risk, source identification, and pollution assessment of heavy metals in road dust: a case study in Rafsanjan, SE Iran. Environ Sci Pollut Res Int. 2018; 25: 13382–95. doi: 10.1007/s11356-017-8539-y 28255819

[34] Hakanson L. An ecological risk index for aquatic pollution control. A sedimentological approach. Water Res. 1980; 14: 975–1001.

[35] Duodu GO, Goonetilleke A, Ayoko GA. Comparison of pollution indices for the assessment of heavy metal in Brisbane River sediment. Environ Pollut. 2016; 219: 1077–91. doi: 10.1016/j.envpol.2016.09.008 27614908

[36] Han YM, Du PX, Cao JJ, Posmentier ES. Multivariate analysis of heavy metal contamination in urban dusts of Xi’an, Central China. Sci Total Environ. 2006; 355: 176–86. doi: 10.1016/j.scitotenv.2005.02.026 15885748

[37] Iran Environmental Protection Organization. Soil resource quality standards and guidelines, Deputy Minister of Human Environment. Water and Soil Office. 2013; 166p. (In Persian).

[38] Bai J, Wang Q, Zhang K, Cui B, Liu X, Huang L. et al. Trace element contaminations of roadside soils from two cultivated wetlands after abandonment in a typical plateau lakeshore, China. Stoch Environ Res Risk Assess. 2011; 25: 91–7.

[39] Yang QQ, Li ZY, Lu XN, Duan QN, Huang L, Bi J. 2018, A review of soil heavy metal pollution from industrial and agricultural regions in China: Pollution and risk assessment. Sci Total Environ. 2018; 642: 690–700. doi: 10.1016/j.scitotenv.2018.06.068 29909337

[40] Jaffar STA, Luo F, Ye R, Younas H, Hu XF, Chen LZ. The extent of heavy metal pollution and their potential health risk in topsoils of the massively urbanized district of Shanghai. Arch Environ Contam Toxicol. 2017; 73: 362–76. doi: 10.1007/s00244-017-0433-6 28718158

[41] Cai QY, Mo CH, Li HQ, Lü H, Zeng QY, Li Y. et al. Heavy metal contamination of urban soils and dusts in Guangzhou, South China. Environ Monit Assess. 2012; 185:1095–106. doi: 10.1007/s10661-012-2617-x 22592780

[42] Xu P, Chen Y, He S, Chen W, Wu L, Xu D. et al. A follow-up study on the characterization and health risk assessment of heavy metals in ambient air particles emitted from a municipal waste incinerator in Zhejiang, China. Chemosphere. 2020; 246: 125777. doi: 10.1016/j.chemosphere.2019.125777 31901657

[43] Lee CS, Li X, Shi W, Cheung SC, Thornton I. Metal contamination in urban, suburban, and country park soils of Hong Kong: A study based on GIS and multivariate statistics. Sci Total Environ. 2006; 356: 45–61. doi: 10.1016/j.scitotenv.2005.03.024 15913711

[44] Guney M, Onay TT, Copty NK. Impact of overland traffic on heavy metal levels inhighway dust and soils of Istanbul, Turkey. Environ Monit Assess. 2010; 164:101–10. doi: 10.1007/s10661-009-0878-9 19347593

[45] Jiries AG, Hussein HH, Halash Z. The quality of water and sediments of street runoff in Amman, Jordan. Hydrological Processes. 2001; 15:815–24.

[46] De Miguel E, Llamas JF, Chacon E, Berg T, Larssen S, Royset O. et al. Origin and patterns of distribution of trace elements in street dust: Unleaded petrol and urban lead. Atmos Environ. 1997; 31: 2733–40.

[47] Mielke HW, Gonzales CR, Smith MK, Mielke PW. The urban environment and children’s health: Soils as an integrator of lead, zinc, and cadmium in New Orleans, Louisiana, U.S.A. Environ Res. 1999; 81: 117–29. doi: 10.1006/enrs.1999.3966 10433843

[48] Facchinelli A, Sacchi E, Mallen L. Multivariate statistical and GIS-based approach to identify heavy metal sources in soils. Environ Pollut. 2001; 114:313–24. doi: 10.1016/s0269-7491(00)00243-8 11584630

[49] Zong Y, Xiao Q, Lu S. Magnetic signature and source identification of heavy metal contamination in urban soils of steel industrial city, Northeast China. J. soils sediments. 2017; 17:190–203.

[50] De Miguel E, Llamas JF, Chacon E, Mazadiego LF. Sources and pathways of trace elements in urban environments: a multi-elemental qualitative approach. Sci Total Environ. 1999; 235: 355–7.

[51] Guo G, Wu F, Xie F, Zhang R. Spatial distribution and pollution assessment of heavy metals in urban soils from southwest China. J Environ. Sci. 2012; 24: 410–8. doi: 10.1016/s1001-0742(11)60762-6 22655353

[52] Luo XS, Xue Y, Wang YL, Cang L, Xu B, Ding J. Source identification and apportionment of heavy metals in urban soil profiles. Chemosphere. 2015; 127:152–7. doi: 10.1016/j.chemosphere.2015.01.048 25698100

[53] Nicholson FA, Smith SR, Alloway BJ, Carlton-Smith C, Chambers B. 2003, An inventory of heavy metals inputs to agricultural soils in England and Wales. Sci Total Environ. 2003; 311: 205–19. doi: 10.1016/S0048-9697(03)00139-6 12826393

[54] Khadem-Moghadam N, Golchin A. Risk Assessment of Contamination of the Country’s Soil and Water Resources with Arsenic. Iranian Journal of Soil and Water Research. 2019; 50:1612–17. (In Persian with English abstract).

